# Supplementary material for: Theory-Guided Design of a Method to Obtain Competitive Balance between U(VI) Adsorption and Swaying Zwitterion-Induced Fouling Resistance on Natural Hemp Fibers
Source: Int J Mol Sci. 2022 Jun 10;23(12):6517. doi: 10.3390/ijms23126517 (PMC9223365; doi:10.3390/ijms23126517)
Supplement: Supplementary file 1 [file ijms-23-06517-s001.zip › ijms-1717977-supplementary.pdf]

Theory-Guided Design of Competitive Balance between U(VI) Adsorption and Swaying Zwitterion Induced Fouling Resistance on Natural Hemp Fibers

Huiquan Gu,[1,2] Jing Yu,[1,2]* Hongsen Zhang,[1,2] Gaohui Sun,[2] Rumin Li,[1,2] Peili Liu,[1,2] Ying Li,[3] Jun Wang[1,2]*

1 Key Laboratory of Superlight Materials and Surface Technology, Ministry of Education, Harbin Engi-neering University, Harbin 150001, China

2 College of Materials Science and Chemical Engineering, Harbin Engineering University, Harbin 150001, China

3 Laboratory of Theoretical and Computational Chemistry, College of Chemistry, Jilin University, Changchun 130023, China.

Corresponding author: Jing Yu

E-mail address: jing.yu@hrbeu.edu.cn

Corresponding author: Jun Wang

E-mail address: zhqw1888@sohu.com

1. Materials

Hemp fibers (HF) was purchased from Shenyang Beijiang Hemp Industry Development co., LTD. Hexamethylene diisocyanate (HDI), N,N-dimethylethanolamine (DMEA), and 1,4-butyrolactone were purchased from Aladdin. N,N-dimethylformamide (DMF) and tetrabutylammonium bromide were purchased from Tianjin Guangfu Fine Chemical Reaserch Institute. Acrylonitrile was brought from Sinopharm Chemical Reagent Co., Ltd. Dibutyltin dilaurate (DBTDL) was purchased from Tokyo Chemical Industry. The purity of all the chemicals above was A.R. The sea salt was purchased from Haiyang Guangzhou aquarium Technology Co., Ltd. The HNO_3 (UPS, 68%) was obtained from Suzhou Crystal clear chemical Co., Ltd. The water used in all experiments was deionized water. *Escherichia coli* (*E. coli*) and *Staphylococcus aureus* (*S. aureus*) were purchased from Shanghai Preservation Biotechnology Center. Marine bacteria were extracted from the sea area of Dalian city, Liaoning province, China.

2. Instrumentations

The morphology and structure of the microspheres were characterized by a field emission scanning electron microscope (SEM, Hitachi S4800). Fourier-transform infrared (FT-IR) spectra of the microspheres were recorded to analyze the surface characteristics of the nanocarriers on an AVATAR 360 FTIR spectrophotometer in the 400-4000 cm^{-1} region by using the KBr-disk method. X-ray photoelectron spectroscopy (XPS) measurements were performed using a PHI 5700 ESCA spectrometer with Al KR radiation (h_v= 1486.6 eV). The algae cell concentration was observed via an optical microscope (Leica DML 300B, Germany). Contact angle tester (4 μL of deionized water on the surface of the adsorbent OCA100, German dataphysics). Inductively coupled plasma-atomic

emission spectroscopy (ICP-AES, Optima-7000DV) was used to analyse the concentration of uranium (VI) and the trace U(VI) ions concentration was measured by Inductive Coupled Plasma (ICP, Bruker 820-MS).

3. Antifouling experiments

Three kinds of diatom in marine were selected to evaluate the biofouling resistance of HF-based materials, named *Nitzschia closterium* (*N.* c*losterium*), *Phaeodactylum tricornutum* (*P. tricornutum*) and *Halamphora sp.*, pruched from the Center for Collections of Marine Algae of Xiamen University. Precisely, diatoms were grown and cultivated in F/2 medium without aeration at 21 ± 2 °C with a 12 h light and 12 h dark (L/D) cycle of fluorescent illumination and stirred every 12 h. After the number of cells is above 10^5 cells·mL^{-1}, 50 mL of diatoms suspension and 20 mg HF-based adsorbents were added into conical flasks. The conical flasks were incubated and kept for further analysis after immersed for 2 and 7 days in a biochemical incubator. To determine the anti-adhesion properties of the samples, the experiments were carried out by the optical microscope and observing the attachment of diatoms on the surface of the adsorbents via fluorescence microscope.

Three kinds of bacterials were selected evaluate the biofouling resistance of HF-based materials, named *Staphylococcus aureus (S. aureus)*, *Escherichia coli* (*E. coli*) and marine bacteria. HF-based materials were firstly sterilized by a UV lamp (20 W, 253.7 nm) for 30 min and then placed in triangle bottles, which contained 50 mL Luria-Bertani (LB) liquid culture medium and diluted *E. coli* and *S.aureus* cells with predetermined concentration using the standard serial dilution method. Then the LB liquid culture medium was incubated for 12 h at 37 °C. Then, each HF-based materials was taken out of the liquid culture medium and the *E. coli* and *S. aureus* cells were separated from the

HF-based materials by 10 mL LB liquid culture medium, respectively. The 10 mL LB liquid culture medium with detached *E. coli* and *S. aureus* cells were collected and diluted to 0.1% of the original content. 10 μL of the diluted liquid culture medium was then uniformly scraped onto an LB solid culture medium and incubated for 24 h at 37 °C. The test method for marine bacteria was the same, except that the culture time of marine bacteria was 3 days, and the culture temperature was 25 °C.

4. Batch adsorption experiments

SI.4.1 Adsorption experiments

In the adsorption experiment, 0.02 g of HFAO, HFAC or HFAS was added to a 0.05 L of pH-adjusted $UO_2(NO_3)_2{\cdot}6H_2O$ solution by using 0.5 M HNO_3 and/or saturated Na_2CO_3 at 25 °C, respectively. After the adsorption processes, the conical flasks were allowed to stand for a few minutes and the supernatant solutions were analysed by ICP-AES, followed by calculation with Equation S1 to get the adsorbed amount of U(VI) ions. In the pH experiments, the range of solution pH is 4.0-9.0. And pH=8.3 was used to simulated the pH of seawater instead of 8.0.

As the sorption kinetics govern the residence time of the sorption reaction and determine the solute uptake rate or the efficiency of the reaction, the following pseudo-1st-order, pseudo-2nd-order and Weber-Morris (W-M) models are employed to interpret the mechanism controlling the sorption process. The linear form of the two models can be expressed by the following:

$$Q_e=(C_0\text{-}C_e)\cdot\frac{V}{m} \quad \text{(S1)}$$

$$\ln\left(Q_e\text{-}Q_t\right)=\ln Q_e\text{-}k_1 t \quad \text{(S2)}$$

$$\frac{t}{Q_t}=\frac{1}{k_2 Q_e^2}+\frac{1}{Q_e}t \quad \text{(S3)}$$

$$Q_e = K_{ip}\sqrt{t} + C \quad \text{(S4)}$$

where Q_e is the adsorption capacity of the adsorbent, C_0 and C_e (mg L^{-1}) are the concentrations of U(VI) ions at the initial and equilibrium states, respectively. V (L) is the volume of the solution, and m is the weight of sorbent (g). Q_t and Q_e (mg g^{-1}) are the capacity of U(VI) at time t (min) and at equilibrium, K_{ip} is internal diffusion constant, respectively, and k_1 (min^{-1}) and k_2 (g mg^{-1} min^{-1}) are the respective rate constants.

To further explore the treatment capability of adsorbents, the effect of the initial concentration of U(VI) was investigated; subsequently, the adsorption isotherms were studied to probe the maximum adsorption capacity and the progress of adsorption. The adsorption of U(VI) on the composites increased at 25 °C and the Langmuir, Freundlich and Dubinin-Radushkevich models were applied to simulate experimental data.

$$\frac{C_e}{Q_e} = \frac{1}{bQ_m} + \frac{C_e}{Q_m} \quad \text{(S5)}$$

$$\ln Q_e = \ln k + \frac{1}{n}\ln C_e \quad \text{(S6)}$$

$$\ln Q_e = \ln Q_m - \beta\varepsilon^2 \quad \text{(S7)}$$

$$\varepsilon = RT\ln(1 + \frac{1}{C_e}) \quad \text{(S8)}$$

Where C_e (mg L^{-1}) is the equilibrated U(VI) concentration, Q_e (mg g^{-1}) is the amount of U(VI) adsorbed on the capacity of the adsorbent at equilibrium. K (L mg^{-1}) is a Langmuir constant related to the energy of the adsorbent and Q_m (mg g^{-1}) is the saturation capacity at complete monolayer coverage. β is the activity coefficient and ε is the Polanyi potential.

SI.4.2 Adsorption-desorption cycle experiments of HF-based adsorbents

In a typical experiment, 0.02 g of adsorbent after adsorbing U(VI) ions was added into 0.05 L 0.1 mol·L^{-1} elution (HNO_3, citric acid (CA), $EDTANa_2$, $NaHCO_3$ and NaOH). The flasks were stirred at

room temperature, and then the solid phase was separated from the solution by filtration. The elution was analyzed with ICP-AES to obtain the concentration of U(VI) ions.

SI.4.3 Adsorption in co-existing ions solution, U(VI)-spiked *N. closterium*, *Halamphora sp.* and *P. tricornutum*, and in real seawater

The co-existing ions solution containing U(VI), V(V), Fe(III), Co(II), Ni(II), Cu(II), Zn(II), and Pb(II) was prepared by previous report by replacing seawater with sea salt solution. The concentration of 8 ions in real seawater was also cited. The above cultivated *N. closterium*, *Halamphora sp.* and *P. tricornutum* were spiked by standard U(VI) solution to 1 $mg{\cdot}L^{-1}$. 20 mg adsorbents and 50 mL U(VI)-spiked *N. closterium*, *Halamphora sp.* and *P. tricornutum* solution was put together and shaken for 48 h. The three HF-based adsorbents were placed at 39°13′ E and 122°45′ N, Yellow Sea, China from 10 April to 14 May, 2021 (35 days). After that, the adsorbents were digested by HNO_3. The adsorption capacity of each element of each adsorbent was tested by ICP-MS.

5. Simulation details

The MD simulations were performed with the GRMOACS package. HF-based models and water mixtures were prepared in a cubic box with periodic boundary conditions. The periodic cube box (PCB) was used with the size of 6.0 × 6.0 × 6.0 nm^3. The simple point charge (SPC) model was used to describe water molecules. The parameters used for the model were taken from the OPLS force field. Before starting MD simulation, energy minimization was employed to ensure that the system has no steric clashes or inappropriate geometry. Then, NVT (constant Number of particles, Volume, and Temperature) ensemble was used to achieve the pre-equilibrium. The systems were heated to the target temperature of 298.15 K by using NVT simulations for a total of 1 ns. Finally, the MD production run up to 50 ns was performed to provide configurations for analysis. In all simulations, the V-rescale thermostat algorithm was used to keep the temperature constant at 298.15 K. LINCS algorithm was adopted to constrain bond lengths. The Lennard-Jones interactions were cut off at 0.8 nm for the nonbonded potential. Electrostatics interactions calculated using the particle mesh Ewald (PME) method and the periodic boundary conditions were used throughout. VMD 1.9.3 was used to perform graphs.

6. DFT calculation method

The coordination mode and interaction energy between UO_2^{2+} and the functional moiety were studied by density functional theory (DFT) using the "Gaussian 09 code". The UO_2^{2+} and amidoxime ligand were optimized fully at the B3LYP level. For all the ligand atoms, including C, N, O, and H, the 6-31g basis set was used. For UO_2^{2+}, the SDD basis set was used. The adsorption energy (E_{ads}) of the adsorbents' surface species was calculated by using Equation (S9).

$$E_{ads} = E_{total} - E_{surface} - E_{species} \quad \text{(S9)}$$

wherein, E_{total}, $E_{surface}$ and $E_{species}$ are the total energy of the coordination species, the energy of the empty surface and the energy of rest species, respectively.

7. Tables and Figures

Table S1. XPS analysis of HFAO, HFCB and HFSB

Materials	C (%)	N (%)	O (%)	S (%)
HFAO	65.62	7.01	27.36	-
HFAC	68.59	16	15.41	-
HFAS	64.09	11.76	21.45	2.71

Table S2. Water contact angles of HF-based adsorbents

Materials	Left (º)	Right (º)
HF	74.2	74.8
HFAO	82.4	81.4
HFAC	76.5	77.6
HFAS	68.3	69.1

Table S3. Kinetic parameter for U(VI) adsorption onto HFAO, HFAC and HFAS

Materials	pH	$Q_{e, exp}$ (mg·g^{-1})	Pseudo-1st-order $Q_{e, cal}$ (mg·g^{-1})	k_1 (min^{-1})	R^2	Pseudo-2nd-order $Q_{e, cal}$ (mg·g^{-1})	k_2 (g·mg^{-1}·min^{-1})	R^2
HFAO	8.3	56.63 ± 2.35	41.18±3.12	1.67*10^{-2}	0.8817	56.45±2.27	2.50*10^{-3}	0.9920
HFAC	8.3	59.13 ± 2.82	23.78±4.97	8.19*10^{-3}	0.9686	58.67±1.63	5.44*10^{-3}	0.9962
HFAS	8.3	79.15 ± 2.29	43.25±6.18	7.21*10^{-3}	0.8273	79.13±1.63	5.68*10^{-3}	0.9979
HFAO	5.0	102.31 ± 5.10	91.15±4.31	2.05*10^{-2}	0.9366	109.39±4.72	5.30*10^{-4}	0.9908
HFAC	5.0	111.78 ± 2.78	87.09±8.51	2.06*10^{-2}	0.8325	113.10±4.95	8.59*10^{-4}	0.9905
HFAS	7.0	116.21 ± 2.57	79.24±8.96	1.61*10^{-2}	0.6874	116.48±3.81	1.88*10^{-3}	0.9947

Table S4. W-M parameters for U(VI) adsorption onto HFAO, HFAC and HFAS

Materials		The 1st step			The 2nd step			The 3rd step		
	pH	R_1^2	K_{ip1}	C_1	R_2^2	K_{ip2}	C_2	R_3^2	K_{ip3}	C_3
HFAO	8.3	0.9863	6.21	6.61	0.9925	2.10	28.60	0.7674	0.15	53.89
HFAC	8.3	0.9769	5.83	15.49	0.8667	1.54	40.20	0.6309	0.25	54.54
HFAS	8.3	0.9699	10.37	20.90	0.9513	3.71	45.55	0.6582	0.25	75.07
HFAO	5.0	[illegible]	12.12	-2.38	0.9834	5.83	33.55	0.9958	0.24	98.56
HFAC	5.0	0.9828	13.46	4.01	0.8754	5.12	53.61	0.9741	0.46	103.01
HFAS	7.0	0.9724	14.68	14.81	0.9646	6.97	50.72	0.8878	0.23	111.53

Table S5. Isotherm parameters for U(VI) adsorption onto HFAO, HFAC and HFAS

Materials	pH	$Q_{m, exp}$ ($mg \cdot g^{-1}$)	Freundlich			Langmuir			D-R
			K ($L \cdot g^{-1}$)	n	R^2	$Q_{m, cal}$ ($mg \cdot g^{-1}$)	b ($L \cdot mg^{-1}$)	R^2	R^2
HFAO	8.3	80.78±5.30	16.68	3.32	0.8143	89.55±4.34	0.039	0.9930	0.9372
HFAC	8.3	91.73±3.16	23.66	3.92	0.8299	100.44±2.71	0.045	0.9978	0.8803
HFAS	8.3	111.96±6.66	46.98	6.06	0.7688	115.02±4.32	0.107	0.9958	0.8510
HFAO	5.0	330.42±9.20	56.74	2.67	0.9649	359.83±9.22	0.073	0.9980	0.5561
HFAC	5.0	348.03±14.55	76.37	3.06	0.9442	368.07±17.66	0.121	0.9931	0.9334
HFAS	7.0	367.03±6.94	87.77	3.33	0.9619	383.39±13.77	0.142	0.9961	0.9618

Table S6. Binding energies and bond lengths of the six DFT-calculated structures

Models	E_{ads} (eV)	bond length (Å)					
		1	2	3	4	5	6
$AO(UO_2)(CO_3)(H_2O)$	-38.83	2.19	2.45	2.44	2.35	-	-
η^2-$AO(UO_2)(CO_3)(H_2O)_2$-1	-36.66	2.46	2.33	2.28	2.41	2.49	-
η^2-$AO(UO_2)(CO_3)(H_2O)_2$-2	-36.49	2.40	2.37	2.56	2.39	2.38	2.74
η^2-$AO(UO_2)(CO_3)(H_2O)$	-36.22	2.40	2.32	2.57	2.44	2.32	-
η^2-$AO(UO_2)(CO_3)_2$	-35.67	2.48	2.61	2.40	2.40	2.45	2.58
η^1-$AO(UO_2)(CO_3)_2$	-35.44	2.37	2.40	2.41	2.41	2.50	-

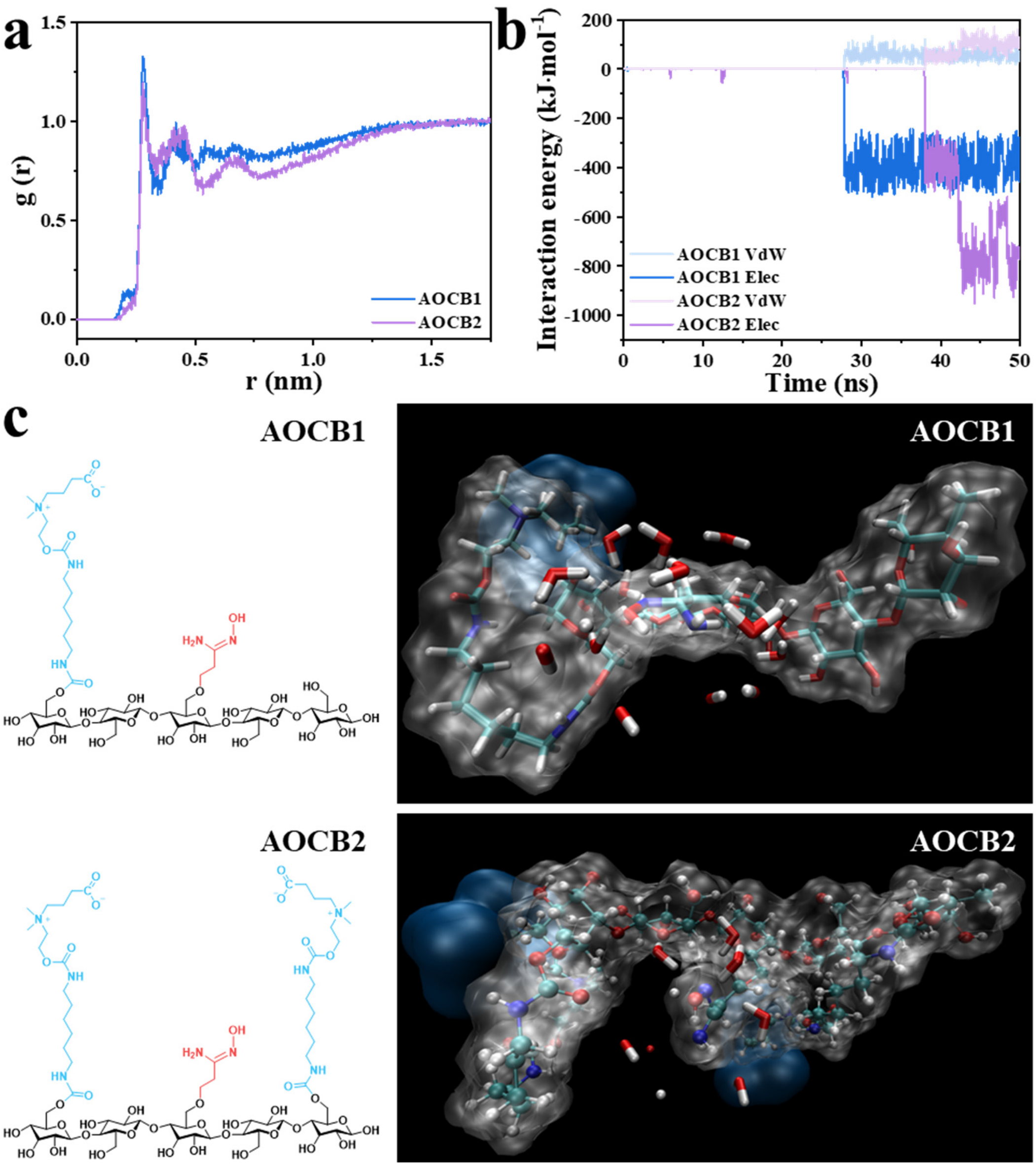

Figure S1. The RDF of water molecules around AO groups (a), the interaction energy between AO groups and U(VI) ions (b), and the disposition of water molecules around AO groups (c) from AOCB1 and AOCB2 (white: H, green: C, blue: N, red: O, the blue cloud stands for hydra-tion layer by terminal COO^- group)

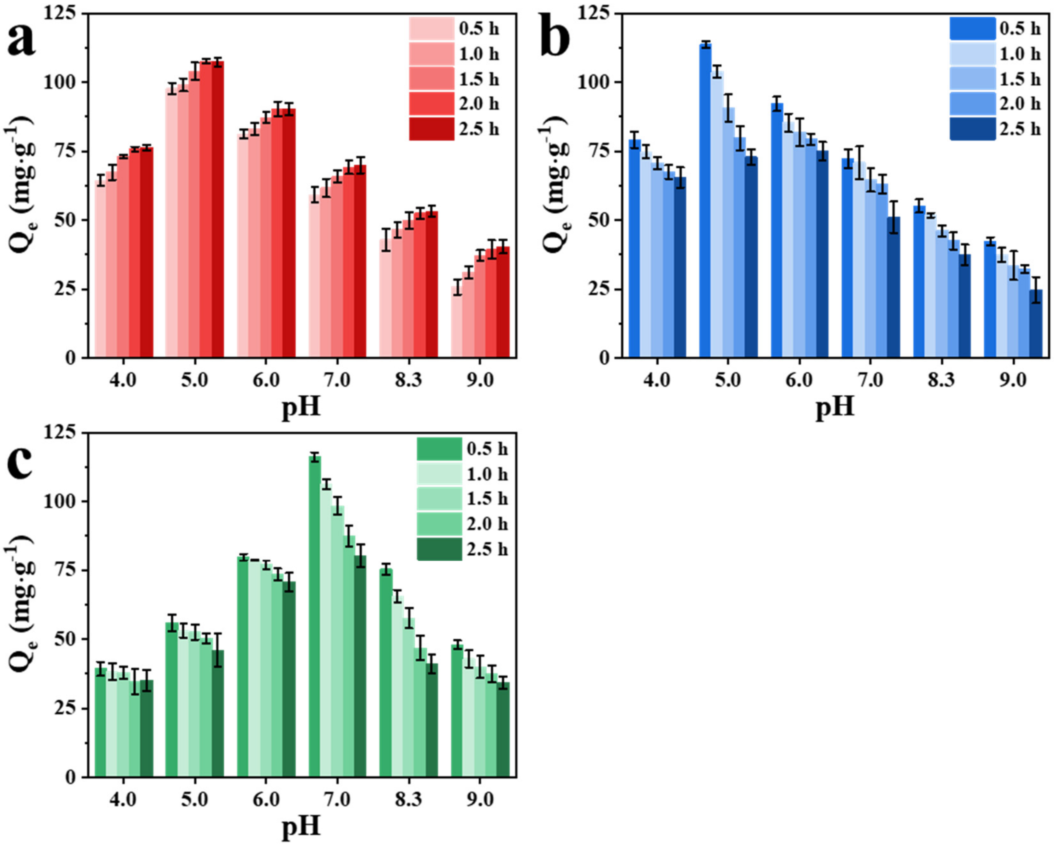

Figure S2. The adsorption capacity of $HFAO_{0.5\text{-}2.5}$ (a), $HFAC_{0.5\text{-}2.5}$ (b), and $HFAS_{0.5\text{-}2.5}$ (c) at different pH

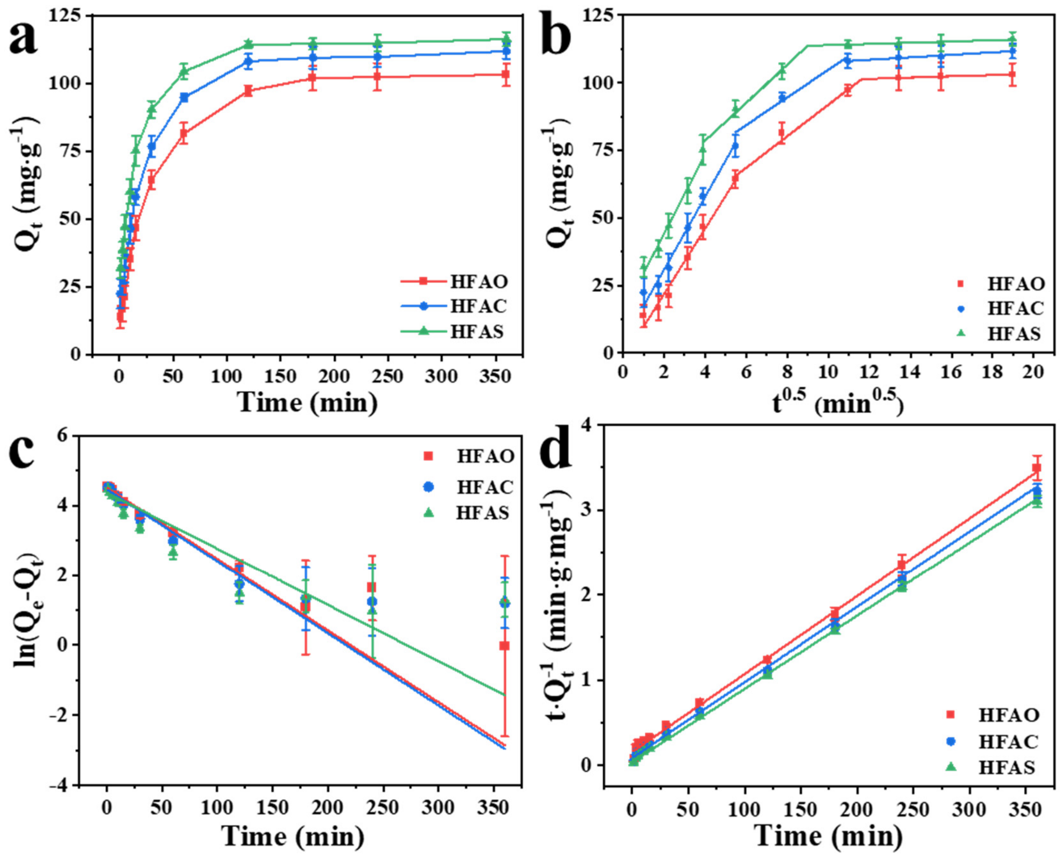

Figure S3. Contact time (a) and kinetics study on HFAO, HFAC and HFAS, W-M (b), pseudo-1st-order (c) and pseudo-2nd-order (d) models linearly fitted curves at optimized pH

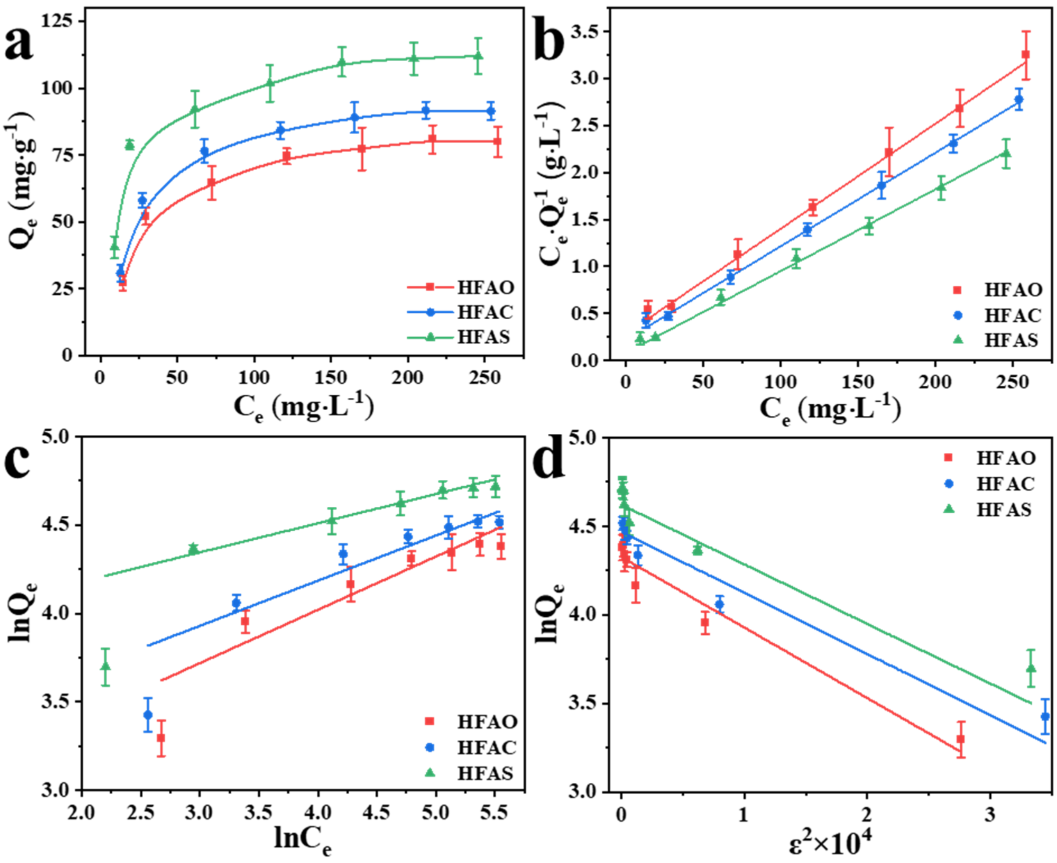

Figure S4. Isotherm (a), Langmuir (b), Freundlich (c), and D-R models (d) for HFAO, HFAC and HFAS at pH = 8.3 under 25 ºC

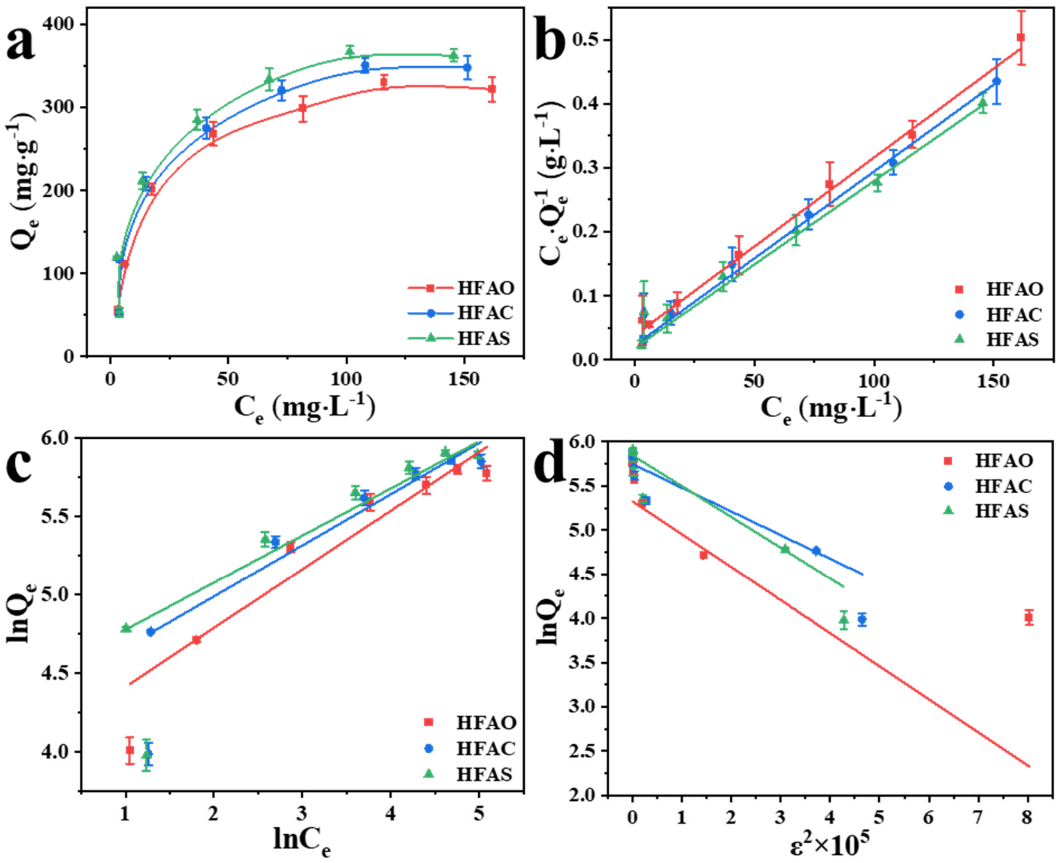

Figure S5. Isotherm (a), Langmuir (b), Freundlich (c), and D-R models (d) for HFAO, HFAC and HFAS at optimized pH under 25 ºC

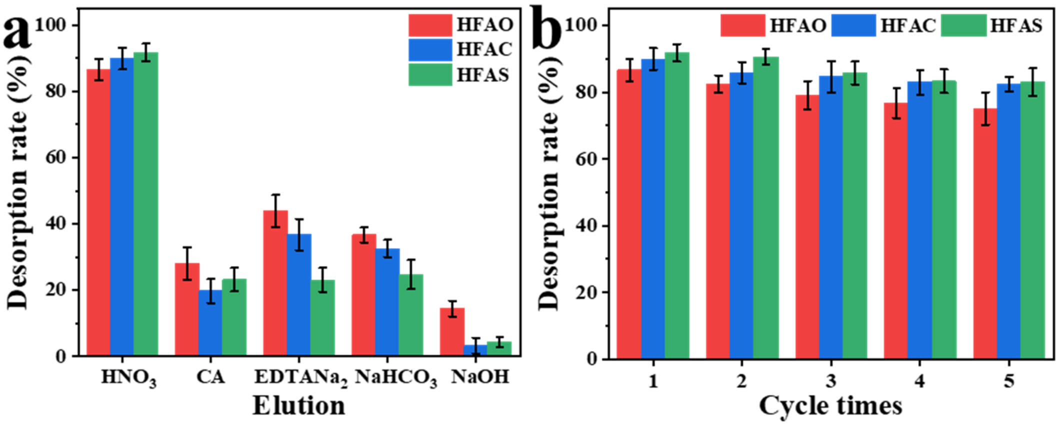

Figure S6. Desorption efficiency of different eluents (a) and removal ratio (b) of HF-based materials under 5 cycles

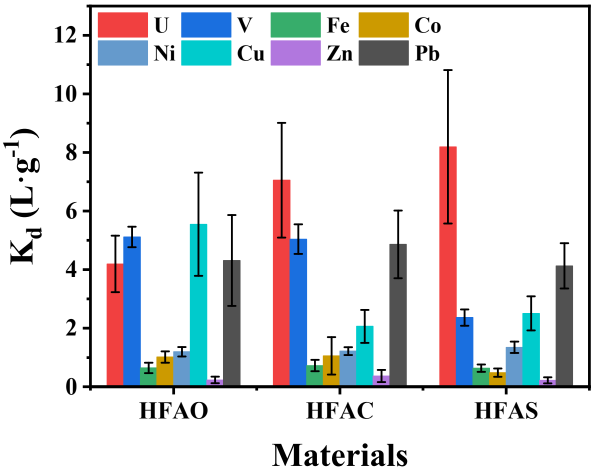

Figure S7. K_d of HFAO, HFAC and HFAS in ion competing solution

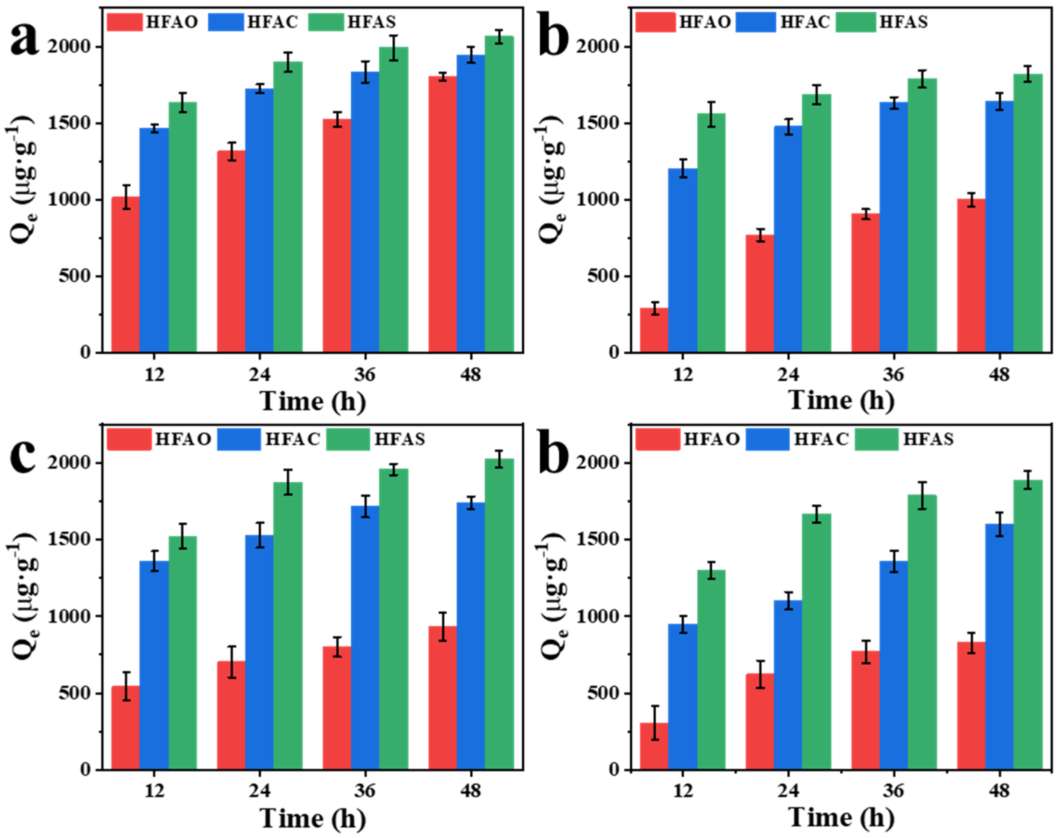

Figure S8. Adsorption capacities of HFAO, HFAC and HFAS in U(VI)-spiked simulated seawater (a) *N. closterium* (b), *P. tricornutum* (c), and *Halamphora sp.* (d) at about 1 mg·L^{-1}

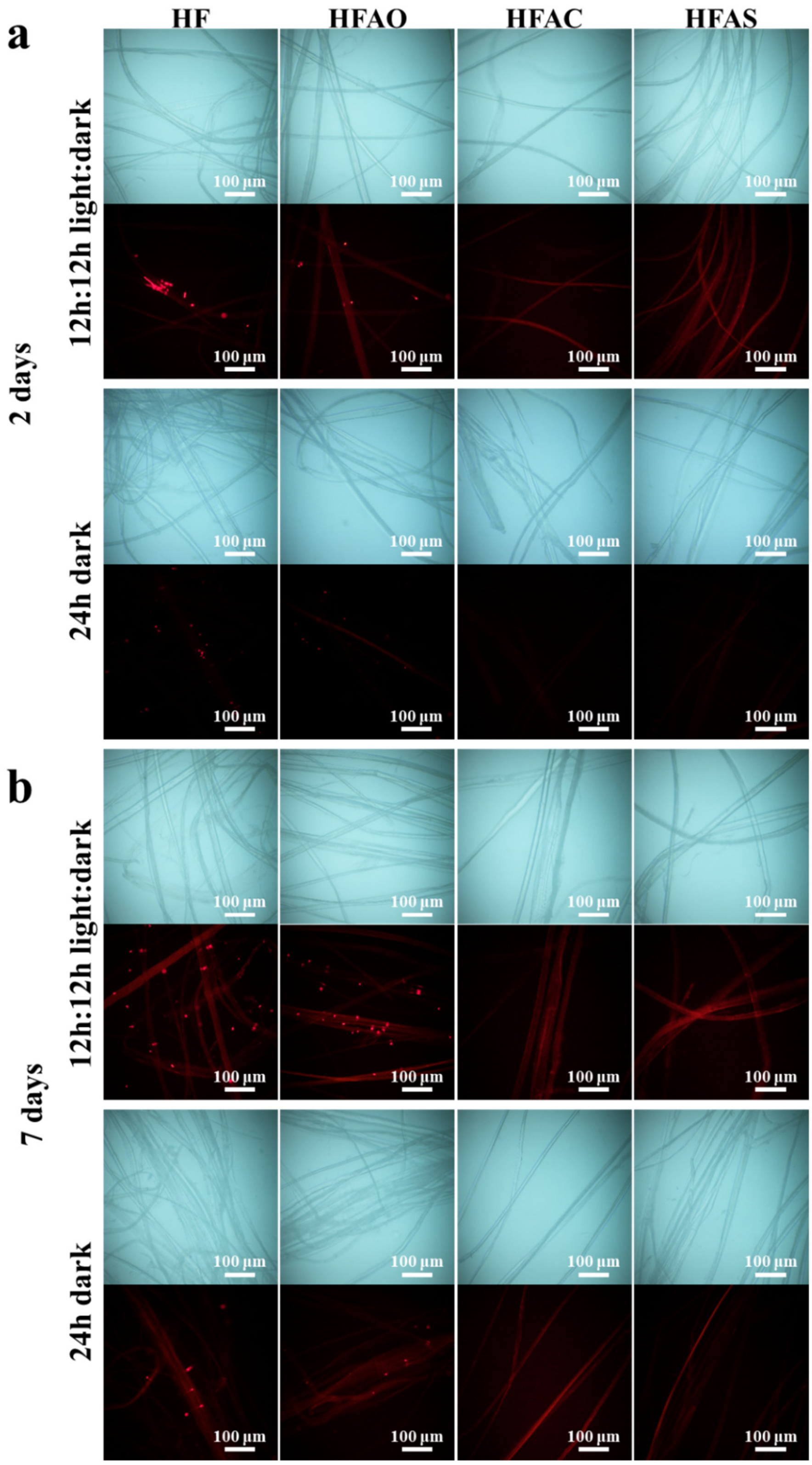

Figure S9. Optical and fluorescence microscopy images of HF, HFAO, HFAC and HFAS after immersed in *N. closterium* for 2 (a) and 7 days (b) under both 12 h light: 12h dark and all dark conditions

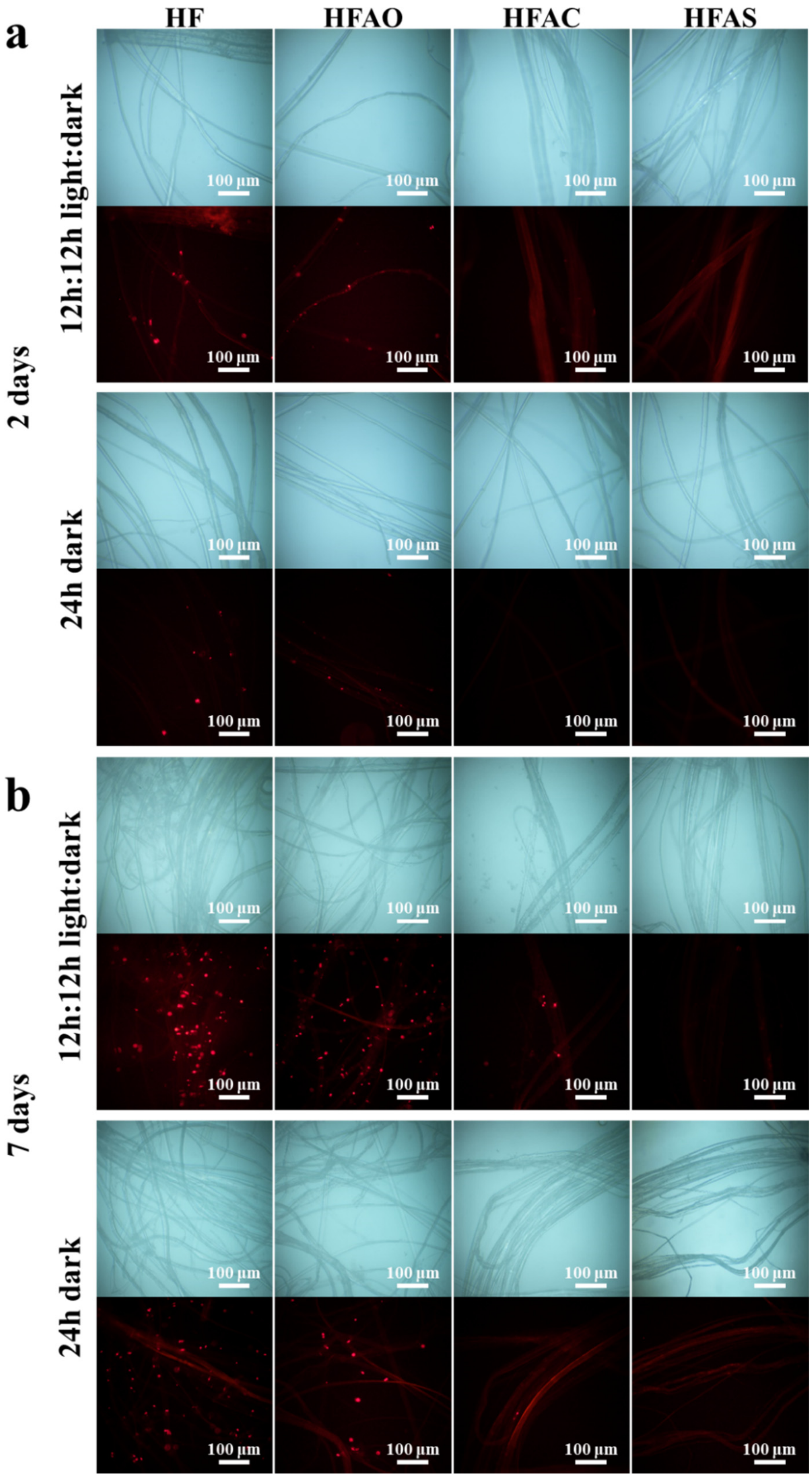

Figure S10. Optical and fluorescence microscopy images of HF, HFAO, HFAC and HFAS after immersed in *P. tricornutum* for 2 (a) and 7 days (b) under both 12 h light: 12h dark and all dark conditions

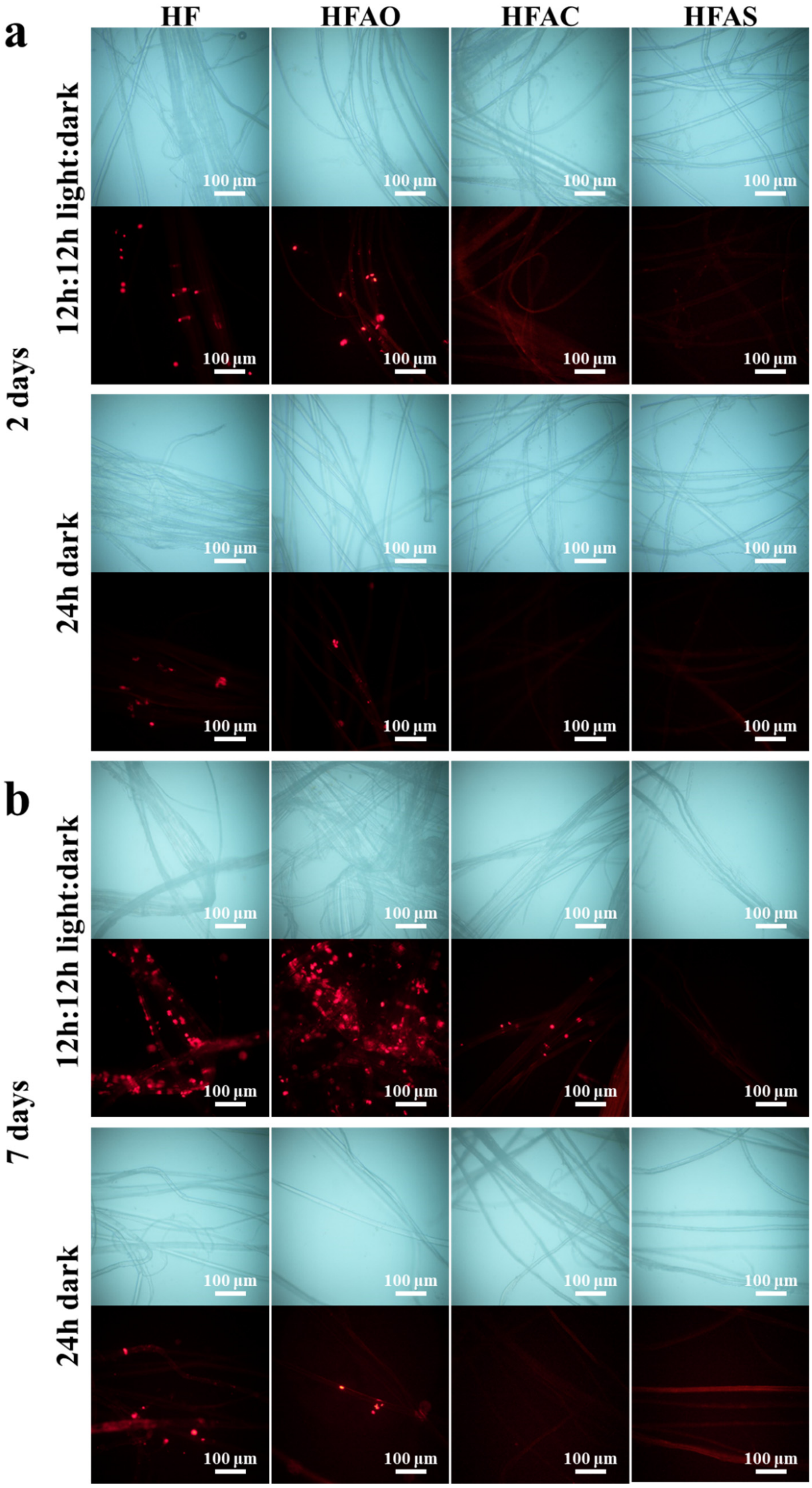

Figure S11. Optical and fluorescence microscopy images of HF, HFAO, HFAC and HFAS after immersed in *Halamphora sp.* for 2 (a) and 7 days (b) under both 12 h light: 12h dark and all dark conditions

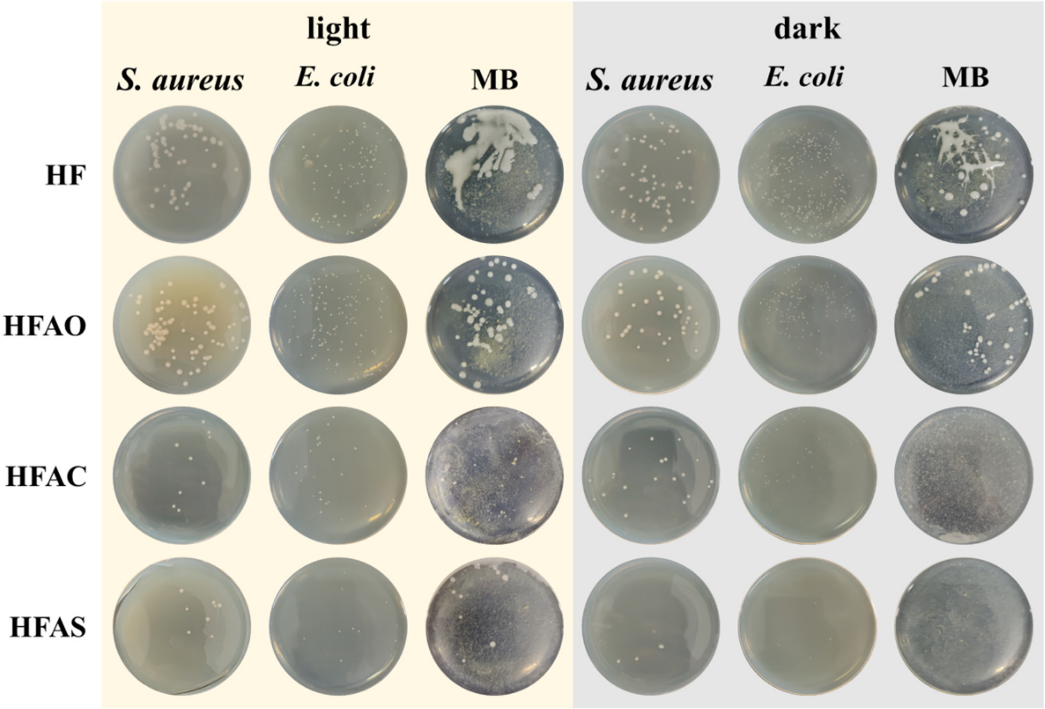

Figure S12. Antifouling property of HF, HFAO, HFAC and HFAS on *S. Aureus*, *E. coli* and marine bacteria under both light and dark conditions
